# Editorial: Immunology of cachexia

**DOI:** 10.3389/fimmu.2023.1339263

**Published:** 2023-12-05

**Authors:** Vijay Kumar, John H. Stewart

**Affiliations:** Department of Surgery, Laboratory of Tumor Immunology and Immunotherapy, Morehouse School of Medicine, Atlanta, GA, United States

**Keywords:** cachexia, immunity, inflammation, NLRP3 inflammasome, cancer, immune-checkpoint inhibitors

Cachexia manifests as a chronic inflammatory condition with profound weight loss that includes muscle wasting/sarcopenia with or without loss of adipose/fat mass (1). It is more common in patients with chronic infections, such as acquired immunodeficiency syndrome (AIDS, a human immunodeficiency virus (HIV)-1 infection) and tuberculosis (caused by *mycobacterium tuberculosis* infection), chronic inflammatory diseases, and many aggressive cancers (gastrointestinal and lung cancers) (2–5). Cancer-associated cachexia (CAC) cannot be easily differentiated from anorexia or other causes of weight or muscle loss. Therefore, CAC is considered as a multifactorial syndrome characterized by ongoing skeletal muscle loss, with or without fat loss and can be partially but not entirely reversed by conventional nutrition support (6). CAC is responsible for 30% of all cancer deaths, although cachexia is not prevalent in all cancer types (7). Furthermore, CAC increases with cancer metastasis, which proves lethal to patients due to failed therapeutic breakthrough to reverse CAC and decreased efficacy of available immunotherapeutics and chemotherapeutics (7, 8). Furthermore, altered immune responses that support tumor growth and metastasis also play critical roles in the development of cachexia. These findings support further investigation (9, 10). Therefore, the current Research Topic entitled “*Immunology of cachexia*” is a step in this direction.

de Cassia Rosa de Jesus et al. discuss the heterogenous expression of the NOD-like receptor (NLR) family pyrin domain containing 3 (NLRP3) that forms inflammasomes upon activation and induces IL-1β and IL-18 secretion to create a pro-inflammatory environment in the adipose tissue (AT) of colorectal cancer (CC) patients. These patients have different comorbidities, including systemic arterial hypertension (seen most frequently). Their study has shown increased systemic inflammation (decreased circulating albumin and increased C-Reactive Protein (CRP) levels) in patients with CC who present with cachexia (defined as per the international consensus criteria) as compared to the control group (1). They further found NLRP3 overexpression in the subcutaneous adipose tissues (scAT) of patients with CC compared to control and weight-stable patients with CC (WSC). They observed similar findings in the visceral adipose tissue (VAT) close to the tumor (peritumoral adipose tissue, PtAT) of patients with CC who present with cachexia. The scAT of patients with CC-associated cachexia and WSC overexpress caspase-1 (CASP-1), a critical component of NLRP3 inflammasome relative to the control group. However, *in vitro* studies associated with lipopolysaccharide (LPS) stimulation in scAT and ptAT have variable results due to dysregulated TLR expression and function. Hence, further research in this area is warranted to establish the immunoregulatory role of NLRP3 inflammasomes in CAC. Furthermore, NLRP3 inflammasome activation plays a role in hypertension therefore further studies in different cells, including immune cells are critical to establish the role of NRLP3 inflammasome activation in CC cachexia without hypertension (11–13).

Sun et al. have shown that the Institute of Cancer Research (ICR) mice develop skeletal muscle loss/atrophy in response to continuous IL-6 infusions in their tibialis anterior muscles via immune receptor activation and metabolic energy reduction. For example, IL-6 infusion downregulates several genes involved in oxidative phosphorylation (OXPHOS) and tricarboxylic acid (TCA) or Krebs cycle and supports aerobic glycolysis to support pro-inflammatory immunometabolic reprograming (14, 15). Furthermore, glycolysis also supports NLRP3 inflammasome activation to aggravate the inflammation and skeletal muscle cell pyroptosis (16, 17). They have further shown that signal transducer and activator of transcription 3 (STAT3), NF-κB, tumor protein 53 (TP53 or p53), and myogenin (MyoG) signaling in response to IL-6 are critical for cachexia-associated muscle atrophy. Hence, IL-6 via glycolysis may activate NLRP3 inflammasome in skeletal muscle and fat cells of patients with cancer to induce cachexia.

Sun et al. have shown the differential expression of necroptosis-related genes (NRGs) in patients with CC and help to classify patients into high and low-risk groups. Furthermore, necroptosis mediates muscle protein degradation in a cachexia model and proteins that signal for necroptosis can activate NLRP3 inflammasome (18–20). Thus, NLRP3 inflammasome activation through different interconnected pro-inflammatory signaling events serves as a critical mediator of the CAC. Hence, future studies that delineate necroptosis and immune reprograming in CAC are needed. Furthermore, the study by Pinci et al. in the Research Topic has indicated that tumor necrosis factor (TNF), released by the activation of a disintegrin and metalloproteinase (ADAM proteases), serves as a necroptosis-associated alarmin. The increased TNF levels support cachexia by increasing the loss of AT and proteolysis and decreasing protein, lipid, and glycogen synthesis, along with its pro-inflammatory action (21). Therefore, TLR and NLRP3 activation-mediated pro-inflammatory events are critical players in the immunopathogenesis of cachexia and need further investigation.

Cunningham et al. investigated the platelet status in cancer cachexia progression by using Apc^Min/+^ mice. This study indicates that platelet numbers increase before cachexia development and can become activated during its progression. In the pre-cachexia stage, these mice showed elevated levels of transforming growth factor β2 (TGFβ2), TGFβ3, Mothers against decapentaplegic homolog 3 or SMAD family member 3 (SMAD3), and IL-1β overexpression in their skeletal muscles. The Apc^Min/+^ mice with severe cachexia overexpressed Ly6G, CD206 (mannose receptor), and IL-10 mRNA. The pre-cachectic Apc^Min/+^ mice show decreased physical activity and anemia that increases with the cachexia severity. Earlier clinical study has indicated a negative association between platelet count and one-year overall survival of patients with cancer cachexia (22). Furthermore, increased platelet count is associated with renal cachexia and cardiovascular mortality in end-stage renal disease patients (23). Hence, understanding the immunological functions of platelets in cachexia is important. For example, NLRP3 inflammasome activation in platelets is a critical pro-inflammatory event for multi-organ injury and platelets also regulate the NRLP3 inflammasome activation in other immune cells, such as macrophages and neutrophils by licensing The NLRP3 transcription (24, 25). Therefore, it will be interesting to observe the platelet mediated NLRP3 inflammasome regulation in myeloid immune cells (MICs), myocytes, and adipocytes of patients with CAC.

Minagawa et al. have shown that deleting the transformed follicular lymphoma (TFL) gene induces extraordinary C-X-C chemokine ligand 13 (CXCL13 or B cell-attracting chemokine 1or BCA-1) secretion and cachexia development in transgenic VavP-*Bcl2* mice, thus causing early death. CXCL13 is a ligand for CXCR5 (Burkitt’s lymphoma receptor 1 or BLR1) and controls B cell development and trafficking (26, 27). Hence, this study indicates that CXCL13 overexpression and cachexia development in lymphoma occur downstream of TFL activity. Further studies will help to understand the immunological role of CXCL13 and TFL in other cancers and associated cachexia. Therefore, innate and adaptive immune components play a critical role in the immunology of CAC depending on the tumor type and stage, comprising a novel therapeutic approach for immunotherapeutic targeting.

Immune checkpoint inhibitors (ICIs) are the latest immunotherapies available to patients with cancer. The review by Li et al. discuss the advanced hepatocellular carcinoma (AHCC) tumor immune microenvironment (TIME) and role of PD-1/PD-L1 checkpoint inhibitors alone or in different combinations along with associated challenges. Furthermore, a clinical case report by Li et al. suggests the efficacy of S-1 and Oxaliplatin (SOX) chemotherapy with anti-PD-1 and invariant natural killer T (iNKT) cell immunotherapies in a patient with stage IV gastric adenocarcinoma with liver metastasis. Patients with Stage IV gastric adenocarcinoma with liver metastasis develop severe cachexia. Therefore, the cachexia index (CXI) can serve as a good prognostic marker in patients with gastric cancer (GC) and CC (28, 29) This is applicable to patients with low CXI, particularly those combined with cachexia, low body mass index (BMI) or advanced stage cancer (28, 29). Therefore, CXI has the potential to serve as a predictive marker for metastatic GC and efficacy of immunotherapeutic agents. Furthermore, Jia et al. in their clinical trial data, have indicated the efficacy of sintilimab in combination with autologous NK cells as a second-line treatment for patients with advanced non-small cell lung cancer (NSCLC). Cachexia is a negative prognostic indicator in patients receiving second line systemic chemotherapy (30). Therefore, it is important to observe the impact of autologous NK cell-based immunotherapy on cachexia in patients with advanced cancers and vice versa.

Cachexia influences the efficacy of ICIs, such as PD-1/PD-L1 inhibitors in advanced cancers. For example, cachexia in patients with advanced NSCLC reduces the efficacy of PD-1/PD-L1 checkpoint inhibitors (31, 32). Therefore, predicting the risk of cachexia development in advanced cancers, including advanced NSCLC and AHCC, before the starting ICIs has a potential to improve clinical outcomes (33, 34).

In the last article of the Research Topic, Robinson et al. have discussed the impact of inflammation and acute phase activation in cancer cachexia. For example, emerging studies indicate the presence of intact regulatory type 2 immunity (abundant IL-33 and eotaxin-2 level) in the visceral adipose tissue (VAT) of experimental and clinical cases of CC and pancreatic cancer-associate cachexia (35). Furthermore, local macrophages in the VAT are also critical for cachexia-induced fat loos in patients with cancer, including HCC (36). Therefore, cachexia, including CAC pathogenesis involves dysregulated immune response and suggests further research to understand the immunology of cachexia and designing novel immune-based biomarkers and therapeutics.

## Author contributions

VK: Conceptualization, Writing – original draft, Writing – review & editing. JS: Writing – review & editing.
